# Gm40600 suppressed SP 2/0 isograft tumor by reducing Blimp1 and Xbp1 proteins

**DOI:** 10.1186/s12885-019-5848-1

**Published:** 2019-07-16

**Authors:** Ying Fang, Ruonan Xu, Bing Zhai, Chunmei Hou, Ning Ma, Liang Wang, Gencheng Han, Zhenyu Jiang, Renxi Wang

**Affiliations:** 1 Department of Rheumatology, First Hospital of Jilin University, Changchun, 130021 China; 2 Laboratory of Immunology, Institute of Basic Medical Sciences, P.O. Box 130 (3), Taiping Road #27, Beijing, 100850 China; 3 College of Life Science and Technology, Xinjiang University, Urumqi, 830046 Xinjiang China; 4 Department of Geriatric Hematology, Chinese PLA General Hospital, Beijing, 100853 China

**Keywords:** Gm40600, Plasma cells, Multiple myeloma, SP 2/0 cells

## Abstract

**Background:**

Multiple myeloma (MM), characterized by cancerous proliferation of plasmablasts (PB) and plasma cells (PC), remains incurable in many patients. Differentially expressed molecules between MM PCs and healthy PCs have been explored in order to identify novel targets for treating MM. In the present study, we searched for novel MM therapeutic targets by comparing mRNA expression patterns between the *Mus musculus* myeloma plasmablast-like SP 2/0 cell line and LPS-induced PB/PC.

**Methods:**

Gene expression profiles of LPS-induced PB/PC and SP 2/0 cells were determined using RNA-sequencing. A predicted gene (Gm40600) was found to be expressed at a low level in SP 2/0 cells. To study the role of Gm40600 in malignant PC, Gm40600 cDNA was cloned into a lentiviral vector (LV201) containing a puromycin selectable marker that was then transfected into SP 2/0 cells. Stable Gm40600-expressing SP 2/0 cells were selected using puromycin. The effect of Gm40600 on SP 2/0 cell proliferation, cell cycle/apoptosis, and tumor progression was assessed by cell counting kit-8 (CCK8), flow cytometry (FACS), and the SP 2/0 isograft mouse model, respectively. The effect of Gm40600 on mRNA and protein expression was evaluated by RNA-sequencing and western blotting, respectively.

**Results:**

We found that SP 2/0 cells expressed lower level of Gm40600 mRNA as compared to LPS-induced PB/PC. Overexpression of Gm40600 significantly suppressed SP 2/0 cell proliferation and isograft tumor progression in an isograft mouse model by promoting apoptosis. In addition, Gm40600 overexpression suppressed transcription of the gene encoding Bcl2. Gm40600 overexpression also reduced the expression of PC-associated transcription factors Blimp1 and Xbp1, which promote transcription of the gene that encodes Bcl2.

**Conclusions:**

Gm40600 reduced SP 2/0 cell proliferation and isograft tumor growth and progression by suppressing Blimp1 and Xbp1-mediated Bcl2 transcription to induce apoptosis. Thus, regulation of a human homolog of Gm40600, or associated factors, may be a potential therapeutic approach for treating MM.

**Electronic supplementary material:**

The online version of this article (10.1186/s12885-019-5848-1) contains supplementary material, which is available to authorized users.

## Background

Each year, deaths due to multiple myeloma (MM) with malignant plasma cells (PC) [1] is approximately 1% of all cancer deaths [2, 3]. This is mainly because of the following two reasons: (1) MM is still incurable in many patients [4, 5]; (2) some patients are refractory to current conventional drugs [6, 7]. Thus, it is urgent to find novel therapeutic way to treat MM [6].

Several reports have shown that MM PC share many common characteristics with healthy PC [2, 3, 8]. Normally, the differentiation of activated B cells into PC is strictly controlled by several genes, including B cell-associated Pax5, germinal center (GC) B cell-associated Bcl6, and PC-specific genes such as IRF4, Prdm1 (Blimp1), and Xbp1 [9, 10]. Bcl2 also promotes B cell differentiation into PC, as Bcl2 with anti-apoptotic activity supports GC B cells and PC survival [11, 12].

As cancerous cells, MM PC express MM-specific molecules that differ from healthy PCs [13–15]. To explore novel MM therapeutic targets, it is necessary to identify molecules that differ between healthy PC and MM PC. Because we found that *Mus musculus* myeloma PB-like SP 2/0 cells (MM PB/PC) expressed a significantly lower level of Gm40600 (a predicted gene) mRNA as compared to LPS-induced PB/PC (normal PB/PC), the effect of Gm40600 on SP 2/0 cell growth was tested.

## Methods

### Mice

Balb/c and CD19^cre^ mice have been previously described [16, 17]. The Floxed Stch (Stch^f/f^) mice in a B6 background were generated by Shanghai Biomodel Organism Science & Technology Development Co.,Ltd. (Shanghai, China). Stch^f/f^ mice were crossed with CD19^cre^ mice to delete Stch in B cells. Gm40600 transgenic mice (cat no. TGB180522CEI02) were purchased from Cyagen Co., Ltd. (Guangzhou, China).

### RNA-sequencing

B220^+^ B cells were sorted from splenocytes of 7- to 9-week female Balb/c, Stch^f/f^, and CD19^cre^Stch^f/f^ mice (3 mice per group) using B220 microbeads (Cat No. 130–049-501, Miltenyi Biotec, Germany), B220^+^ B cells were stimulated with 10 μg/ml LPS (L2630, Sigma, St Louis, MO) for 3 days in vitro as previously described [18]. SP 2/0 cells (ATCC® CRL-1581, Rockville, MD, USA) were thawed, passaged three times, and then cultured for 2 days in fresh medium. RNeasy Mini Kit (Qiagen, Venlo, Netherlands) was used to isolate and purify total RNA from cells. NanoDrop®ND-1000 spectrophotometer and Agilent 2100 Bioanalyzer and RNA 6000 NanoChips (Agilent, Palo Alto, CA, USA) were used to determine RNA concentration and quality, respectively. TruSeq Stranded Total RNA Library Prep Kit with Ribo-Zero Gold (Illumina) was used to prepare Libraries. Transcripts were analyzed by RNA-sequencing (Genewiz Corp., Suzhou, China) using a standard method [18].

### qPCR analysis

Total RNA was extracted from Vector- or Gm40600-expressing SP 2/0 cells, and LPS-stimulated PB/PC with Trizol (Invitrogen Life Technologies). qPCR has been employed using a previous method [18] to quantify mouse Gm40600 gene expression. GAPDH mRNA expression is used to normalized relative mRNA expression that is then calculated relative to mRNA in SP 2/0 cells (set to 1).

### Effect of Gm40600 on SP 2/0 growth

Gm40600 cDNA (accession: XM_011243239) was synthesized by General Biosystems Corp. (Anhui, China) and subcloned into LV201 (Fugene Corp., Guangzhou, China), a lentiviral vector with a puromycin selectable marker. Gm40600-expressing LV201 or control LV201 (empty vector) were transfected into SP 2/0 cells using a previously described method [18, 19]. Puromycin (10 μg/ml; Sigma) was used to select stable transfectants. Counting kit-8 (CCK8), a propidium iodide (PI)/FACS, and the SP 2/0 isograft mouse model were previously described [18, 20, 21] and used to assess the effect of Gm40600 on SP 2/0 cell proliferation, cell cycle, and isograft tumor progression, respectively.

### Gm40600 localization and protein expression

Gm40600 was cloned into EGFP-expressing LV122 (Fugene Corp., Guangzhou, China) to express a Gm40600-EGFP fusion protein. Gm40600-EGFP-expressing LV122 was transfected into SP 2/0 cells using a previously described method [18]. Gm40600-EGFP protein localization was analyzed by immunofluorescence and confocal microscopy according to a previously described method [18]. The effect of Gm40600 on Bcl6, IRF4, Blimp1, Xbp-1, Trp53, and Bcl2 was determined by western blotting using a previously described method [18]. The western blots were probed with rabbit against mouse Bcl6 (sc-858, Santa Cruz Biotech), IRF4 (ab104803), Blimp1 (sc-25,380, Santa Cruz Biotech), Xbp-1 (ab37152, Abcam), Trp53 (#2524, Cell Signaling Tech), Bcl2 (ab59348, Abcam) and β-tubulin (KM9003T, SunGene Biotech) antibodies.

### Bcl2 promoter reporting gene analysis

Lv81/Blimp1 (0.5 μg, cat# EX-Mm24401-Lv81, Fugene Corp., Guangzhou, China), pEZX-PG04.1/Bcl2 promoter (0.5 μg, cat# MPRM19639-PG04, Fugene Corp., Guangzhou, China), and pRLSV-40 vector (0.05 μg, cat# E2231, Promega Corp.) were co-transduced into 4 × 10^5^ 293 T cells according to our previous study [18]. In some experiments, firefly luciferase reporter plasmid pEZX-PG04.1/Bcl2 promoter and renilla luciferase reporter vector pRLSV-40 vector were transduced into stable Gm40600- or vector-expressing SP 2/0 cells. On day 3, 1420 Multilabel Counter (1420 Victor 3, PerkinElmer Corp.) was used to determine the activity of firefly and renilla luciferase.

### Determination of IgM, IgG1, and IgG2a antibody levels by ELISA

B cells from WT and Gm40600 transgenic mice were stimulated for 3 days in vitro with 10 μg/ml LPS. Antibody levels of in the supernatant were determined using mouse IgM, IgG1, and IgG2a ELISA kits (eBiosciences, Cat# 88–50,470-86, 88–50,140-22, and 88–50,420-88, respectively) as the instructions of the manufacturers.

### Statistics

Cellular apoptosis, tumor volume, and tumor weight were compared between the vector alone group and the Gm40600 overexpressing group using the Student’s t test. The change of cell proliferation at three different time points and the percentage of three different cell cycles phases (G1, S, and G2) were compared between the vector alone group and the Gm40600 overexpressing group using two-way ANOVA analysis. SEM was used as mean ± standard error of the mean. *p* < 0.05 value was considered to be statistically significant.

## Results

### SP 2/0 cells express low levels of Gm40600 mRNA

Plasmablasts (PB) induced by LPS [20, 22] and the *Mus musculus* myeloma PB-like SP 2/0 cells were used as normal PB/plasma cells (PC) and MM PB/PC, respectively. Both LPS-stimulated PB/PC and SP 2/0 cells expressed high levels of mRNA corresponding to the following PB/PC-associated transcription factors IRF4, Prdm1 (Blimp1), and Xbp1 (Table 1). Interestingly, as compared to LPS-stimulated PB/PC, SP 2/0 cells expressed a significantly lower level of Gm40600 mRNA (Table 1). Thus, reduction of Gm40600 may promote malignant growth of SP 2/0 cells.

**Table 1:** SP 2/0 cells express low level of Gm40600 mRNA

Gene Symbol	Total exon fragments		FPKM	
	LPS	SP 2/0	LPS	SP 2/0
Pax5	11,625	70	34.34	0.71
Bach2	3318	0	7.92	0
Bcl6	403	230	4.25	5.59
Irf4	9191	5272	12.6	90.41
Prdm1	6055	1233	32.43	18.83
Xbp1	15,347	8127	196.23	293.8
Gm40600	552	4	22.87	0.35

B cells from the splenocytes of three Balb/c mice per group were sorted by B220 microbeads, and stimulated for 3 days in vitro by 10 μg/ml LPS. SP 2/0 cells were thawed, passaged for 3 times and then cultured for 2 days in fresh medium. The transcripts in LPS-stimulated B cells and SP 2/0 cells were determined by RNA-sequencing. Total exon fragments and FPKM (FPKM = total exon fragments / mapped reads (millions) / exon length (KB), Fragments per Kilo bases per Million reads) values of selected genes encoding Pax5, Bach2, Bcl6, Irf4, Prdm1, Xbp1, and Gm40600 are shown

### SP 2/0 cell proliferation was suppressed by Gm40600

**Fig. 1:**
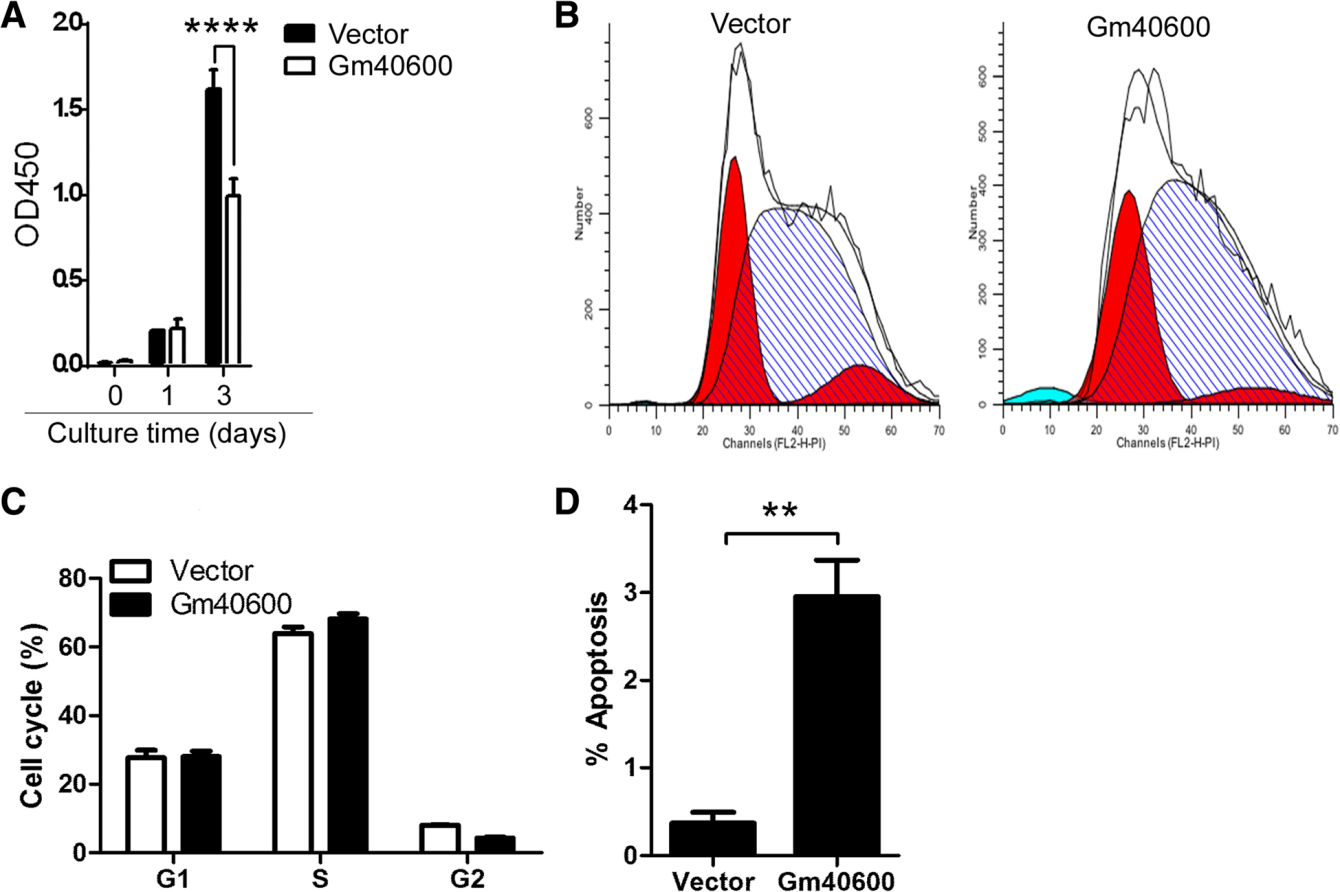
Gm40600 overexpression suppressed proliferation and promoted apoptosis of SP 2/0 cells. **a** Gm40600 overexpression suppressed SP 2/0 cell proliferation. Gm40600- or Vector-expressing SP 2/0 cells were cultured in vitro for 0, 1, and 3 days. CCK8 assay was used to analyze to cell proliferation. **b-d** Gm40600 overexpression affected SP 2/0 cell apoptosis but not cell cycle. Gm40600- or Vector-expressing SP 2/0 cells were cultured in vitro for 2 days. Cells were then stained and analyzed using propidium solution (PI) and flow cytometry (FACS), respectively. FACS profile, cell cycle, and apoptotic cell percentages are shown in (**b**, **c**, and **d**), respectively. **d** Two-tailed Student’s t test and **a**, and **b** two-way ANOVA followed by Bonferroni post-tests to compare each column to control column. Error bars, SEM. **p < 0.01, ****p < 0.0001

To explore the effect of Gm40600 in malignant growth of SP 2/0 cells, an LV201 construct expressing Gm40600 were transfected into SP 2/0 cells. Stable transfectants expressing Gm40600 or Vector were selected by puromycin. qPCR assay demonstrated that Gm40600 mRNA was significantly increased in stably transfected SP 2/0 cells and lower than that in LPS-induced PB/PC (Additional file 1: Figure S1). Critically, CCK8 assay showed that cell number is time-dependently lower in Gm40600-expressing SP 2/0 cells than that in vector-expressing SP 2/0 cells (Fig. 1a). Furthermore, cell cycle and apoptosis analysis suggested that Gm40600 overexpression did not affect the cell cycle but it did promote cell apoptosis (Fig. 1b-d). Collectively, these results demonstrated that Gm40600 significantly suppressed proliferation of SP 2/0 cells by promoting apoptosis.

### Overexpression of Gm40600 suppressed SP 2/0 isograft tumor progression

**Fig. 2:**
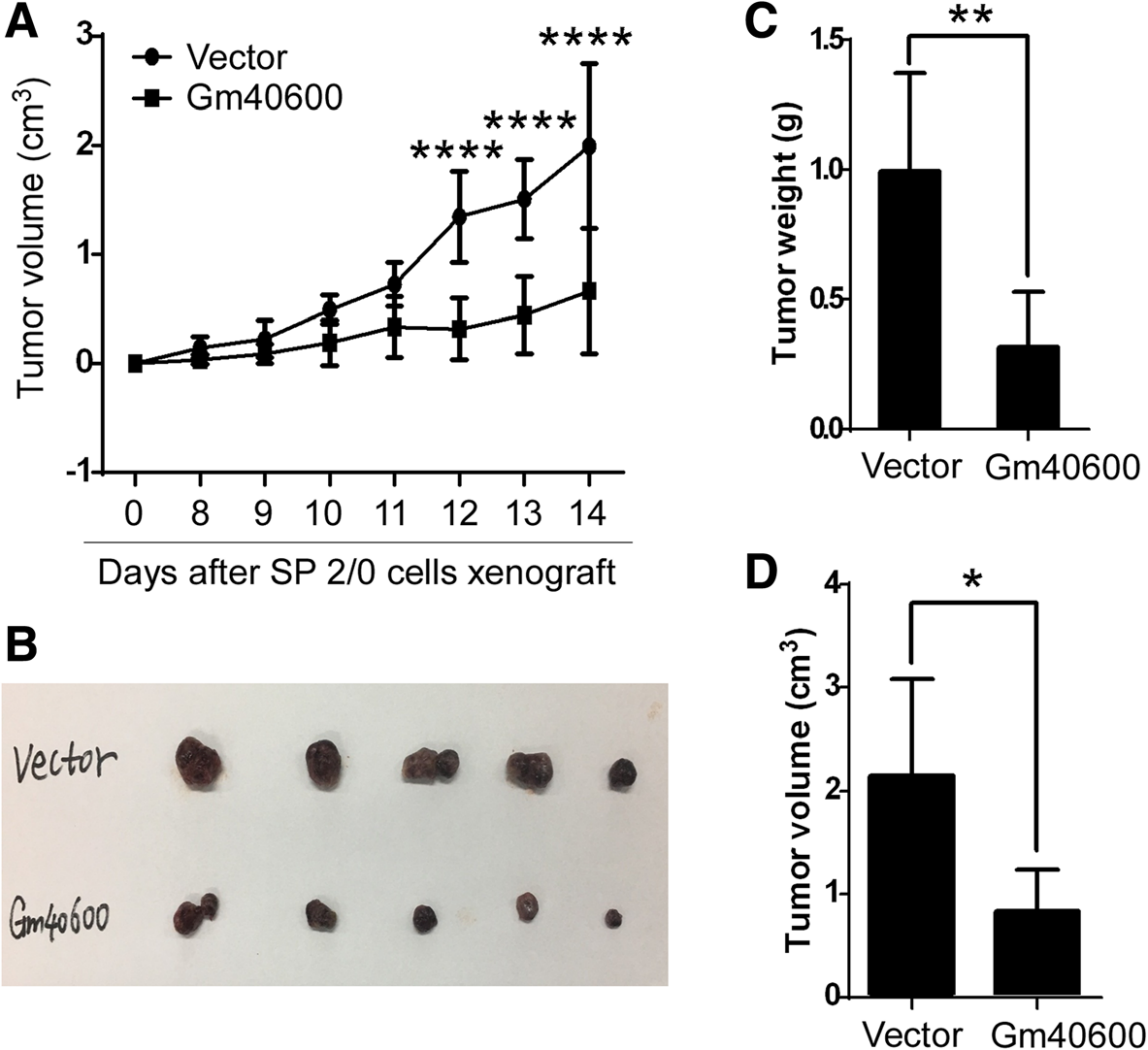
Gm40600 overexpression suppressed tumor progression in the SP 2/0 isograft mouse model. Gm40600- or Vector-expressing SP 2/0 cells were injected subcutaneously into Balb/c mice. **a** Tumor volumes were determined on day 0, 8, 9, 10, 11, 12, 13, and 14 after isograft. On day 14 after the SP 2/0 isograft, images of subcutaneous tumor tissues (**b**), tumor weights (**c**), and tumor volumes (**d**) were recorded. **a**, **c**, and **d** Data from three independent experiments (*n* = 15). **b** Data from one experiment (*n* = 5). Error bars, SEM. Two tailed Student’s t-test, *P < 0.05, **P < 0.01, ****p < 0.0001

The SP 2/0 isograft mouse model [18, 19] was used to assess the effect of Gm40600 on SP 2/0 isograft tumor growth. Gm40600-expressing SP 2/0 cells (5 × 10^6^ cells per mouse) were injected subcutaneously into 8-week-old Balb/c mice (5 mice per group). Tumor volumes were measured on day 0, 8, 9, 10, 11, 12, 13, and 14 after the isograft. Consistent with in vitro data, Gm40600 significantly suppressed SP2/0 isograft tumor progression in a time-dependent fashion (Fig. 2a). Tumors were imaged on day 14 following isograft, which showed that SP 2/0 isograft tumor sizes were decreased by Gm40600 (Fig. 2b). The Gm40600-mediated reduction of the volumes and weights of SP 2/0 isograft tumors was statistically significant (Fig. 2c, d). Collectively, these data demonstrated that Gm40600 suppressed growth and progression of tumors in the SP 2/0 isograft tumor model.

### Gm40600 overexpression reduced Blimp1 and Xbp1 proteins in SP 2/0 cells

As shown above, Gm40600 overexpression promoted SP 2/0 cell apoptosis. Next, RNA-sequencing assay was used to determine which molecule is involved in Gm40600-mediated apoptosis. Gm40600 had no effect on the mRNA levels of Pax5 (mature B cell-associated molecule), Bcl6 and Bach2 (GC B cell-associated molecules), IRF4, Prdm1 (Blimp1) and either unspliced or spliced Xbp1 (PC-associated molecules), Trp53 and Mcl1 (apoptosis-associated molecules) (Table 2). Interestingly, mRNA corresponding to the anti-apoptosis factor Bcl2 was reduced by Gm40600 (Table 2). These results suggest that Gm40600 affected Bcl2 transcription.

**Table 2:** Gm40600 overexpression does not affect the mRNA expression of plasma cells-associated transcription factors and apoptosis-associated molecules

Gene Symbol	Total exon fragments			FPKM		
	Vector	Gm40600	Fold change	Vector	Gm40600	Fold change
Pax5	4.19	12	2.87	0.03	0.09	3
BCL6	171	127	0.74	2.55	2.07	0.81
Bach2	0	0		0	0	
IRF4	14,695	12,090	0.82	163.91	147.68	0.9
Prdm1	2752	2584	0.94	31.65	33.71	1.07
Xbp1 (unspliced)	7731.6	8027.07	1.04	190.96	217.1	1.14
Xbp1 (spliced)	101.4	116.93	1.15	5.23	6.6	1.26
Trp53	4337	5386	1.24	127.9	173.83	1.36
Bcl2	34	18	0.53	0.27	0.14	0.52
Mcl1	21,732	26,477	1.22	406.54	576.35	1.42

The stable Gm40600- and Vector-expressing SP 2/0 cells were cultured for 2 days in fresh medium. The transcripts were determined by RNA-sequencing. Total exon fragments and FPKM (FPKM = total exon fragments / mapped reads (millions) / exon length (KB), Fragments per Kilo bases per Million reads) values of selected genes encoding Pax5, Bach2, Bcl6, Irf4, Prdm1, unspliced and spliced Xbp1, Trp53, Bcl2, and Mcl1, and the fold change of gene expression in Gm40600-expressing SP 2/0 cells to that in vector-expressing SP 2/0 cells are shown

**Fig. 3:**
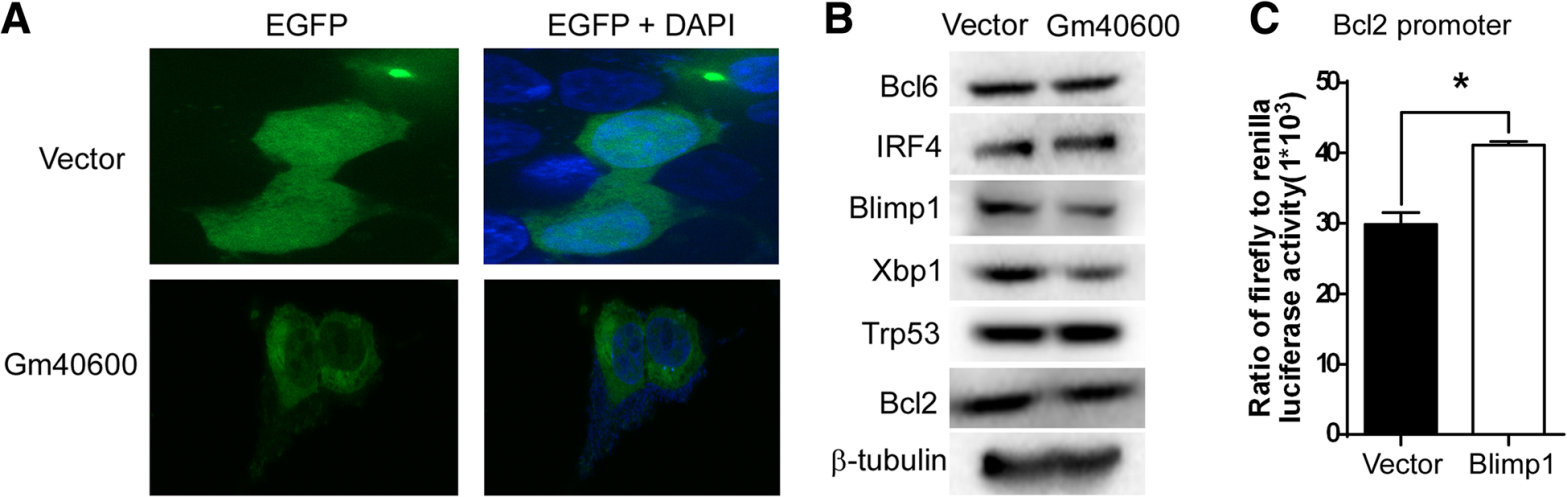
Gm40600 overexpression suppressed the PC-related proteins Blimp1 and Xbp-1 in SP 2/0 cells. **a** Gm40600 localized to the cytoplasm. The stable Gm40600-EGFP (Gm40600)-, or EGFP alone (Vector)-expressing SP 2/0 cells were cultured for 2 days. EGFP were imaged in cells on a GE IN Cell Analyzer 2000. Representative images show cytoplasm localization of Gm40600. **b** Gm40600 overexpression suppressed the PC-related proteins Blimp1 and Xbp-1 in SP 2/0 cells. Gm40600- or vector-expressing SP 2/0 cells described in Figs. 1 and 2 were cultured for 2 days and followed by immunoblot analysis using monoclonal anti-mouse Bcl6, IRF4, Blimp1, Xbp1, Trp53, Bcl2, and β-tubulin antibodies. Results represent three independent experiments. **c** Blimp1 promoted the activation of the Bcl2 promoter. The Blimp1-expressing lentiviral vector LV81 (Blimp1) or empty vector Lv81 (Vector) and the luciferase reporter vector pEZX-PG04.1/Bcl2 promoter (− 1323 ~ + 160 bp) were co-transduced into 293 T cells. Dual luciferase reporter gene expression was analyzed, and the results are shown as the ratio of firefly to renilla luciferase activity. The data represent at least four independent experiments. Error bars, SEM. Two tailed Student’s t-test, *P < 0.05

To explore the mechanism by which Gm40600 regulates transcription, the location of Gm40600 in SP 2/0 cells was examined. SP 2/0 cells expressing either Gm40600-EGFP or EGFP alone were imaged on a GE IN Cell Analyzer 2000. The results demonstrated that EGFP alone was distributed throughout the cells, whereas Gm40600-EGFP was mainly localized to the cytoplasm (Fig. 3a). These results suggest that Gm40600 is not a transcription factor.

Previous studies have shown that Blimp-1 can induce the expression of Xbp-1 [23, 24], which up-regulates the expression of Bcl2 mRNA by directly binding ACTG-CRE sites in the Bcl2 gene promoter [25, 26]. Thus, we propose that Gm40600 affects Bcl2 mRNA expression via Blimp1 and Xbp1. As expected, we found that Gm40600 could reduce Blimp1 and spliced Xbp-1 (~54 kDa) protein in SP 2/0 cells (Fig. 3b, Additional file 1: Figure S2). Accordingly, Gm40600 also reduced expression levels of the anti-apoptosis protein Bcl2 (Fig. 3b) and Bcl2 promoter activation (Additional file 1: Figure S3) in SP 2/0 cells. In addition, Blimp1 significantly activated the Bcl2 gene promoter (Fig. 3c). These results suggest that Gm40600 suppresses Bcl2 gene transcription by reducing Blimp1 and Xbp1 transcription factors.

### Gm40600 overexpression suppressed antibody production in LPS-stimulated primary B cells

To study the role of Gm40600 in normal PC, we found that Stch (a gene coding the heat shock protein family A [Hsp70] member 13) was mainly expressed in PC and a B cell-specific knock-out of Stch did not affect B-cell mature and activation but reduced PC production (data not shown). In this work, Stch deficiency reduced LPS-induced *prdm1* and *xbp1* mRNA by 50% (Table 3). Accordingly, LPS-stimulated Stch^−/−^B expressed almost no Gm40600 (Table 3). Collectively, these data suggest that Gm40600 expression may positively correlate with Prdm1 and Xbp1 expression and involved in nonmalignant PC generation/maintenance.

**Table 3:** Stch deficiency reduced plasma cells and Gm40600 expression induced by LPS

Gene Symbol	Total exon fragments		FPKM	
	Stch^f/f^	CD19^cre^Stch^f/f^	Stch^f/f^	CD19^cre^Stch^f/f^
Pax5	21,163	25,578	135.59	143.21
Bach2	2578	2608	8.17	7.22
Bcl6	660	590	6.38	4.99
Irf4	11,044	9245	81.82	59.86
Prdm1	2294	1288	17.02	8.35
Xbp1	6755	3460	176.95	79.21
Gm40600	391	14	25.96	0.81

B220^+^ B cells from 7 to 9-week-old CD19^cre^Stch^f/f^ mice and their WT littermates (Stch^f/f^) were stimulated for 3 days in vitro with 10 μg/ml LPS. On day 3 following LPS stimulation, the transcripts were determined by RNA-sequencing. Total exon fragments and FPKM (FPKM = total exon fragments / mapped reads (millions) / exon length (KB), Fragments per Kilo bases per Million reads) values of selected genes encoding Pax5, Bach2, Bcl6, Irf4, Prdm1, Xbp1, and Gm40600 are shown

**Fig. 4:**
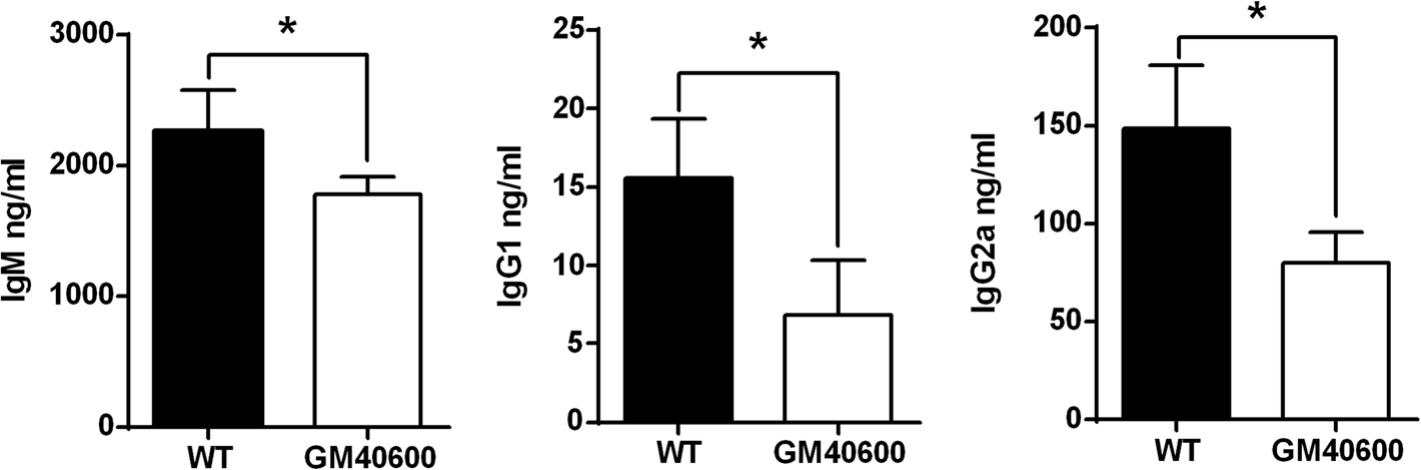
Gm40600 overexpression suppressed antibody production. 10 μg/ml LPS was used to stimulate B cells from the splenocytes of 7- to 9-week-old WT and Gm40600 transgenic mice (three mice per group, total three independent experiments). On 3 days, IgM, IgG1, and IgG2b levels in cell culture supernatant was analyzed using ELISA assay. Error bars, SEM. Two tailed Student’s t-test, *P < 0.05

To further explore the function of Gm40600 in antibody production, Gm40600 transgenic mice were developed. LPS can effectively induce PC production and antibody secretion [20]. Thus, LPS was used to induce antibody production in B cells from wild-type (WT) and Gm40600 transgenic mice. As expected, Gm40600 overexpression suppressed antibody production in LPS-stimulated primary B cells (Fig. 4).

## Discussion

Untill now, the expression pattern of Gm40600 remains unclear and its function is uncharacterized. RNA-sequencing demonstrated that LPS-induced PB/PC expressed a high level of Gm40600 mRNA, whereas cancerous SP 2/0 cells expressed a low level of Gm40600 mRNA (Table 1). In addition, Gm40600 was identified as a suppressive molecule that suppresses cancerous SP 2/0 cell proliferation (Fig. 1a) and isograft tumor progression (Fig. 2). With a high incidence of survival because of usage of these drugs such as bortezomib, thalidomide and lenalidomide [27, 28], MM is still incurable and novel therapeutic strategies are needed [28]. To explore potential therapies, more knowledge of MM biology is needed [28, 29]. This work demonstrated that cancerous SP 2/0 cells expressed very low levels of Gm40600 (Table 1), whereas Gm40600 overexpression suppressed cancerous SP 2/0 cell growth (Figs. 1 and 2). In addition, Gm40600 mRNA was significantly lower in stably transfected SP 2/0 cells than that in LPS-induced PB/PC (Additional file 1: Figure S1). These results suggest that overexpression of Gm40600 in SP 2/0 cells is lower than its physiological expression in LPS-induced PB/PC. This supports that the downregulation of Gm40600 in SP 2/0 cells is a necessary part of tumor development. Thus, it is worth to identify a human homolog for Gm40600 and explore the role of a human homolog of Gm40600 in MM development.

The survival of malignant PC [30], including MM cells [31, 32], was maintained by anti-apoptosis molecules. This inspired researchers to target these anti-apoptotic proteins as a strategy for treating MM [33]. Apoptosis-induced drugs such as Bortezomib have been approved to treat MM [34]. Consistent with these studies, we demonstrated here that Gm40600 overexpression induced SP 2/0 cell apoptosis (Fig. 1b-d).

An important regulator in cell apoptosis is Bcl-2 [35, 36]. Studies have shown that several human tumors aberrantly overexpress Bcl-2, including MM [37, 38]. However, the mechanisms underlying aberrant Bcl-2 expression remain largely unknown [39]. Our data demonstrated that Gm40600 reduced both Bcl2 mRNA and protein in SP 2/0 cells (Table 2, Fig. 3b).

Xbp1 can effectively induce Bcl2 transcription by directly binding to CTG-CRE sites in the Bcl2 gene promoter or indirectly by ESR1 [25, 26, 40]. Our work demonstrated that Gm40600 reduced Bcl2 mRNA (Table 2) by suppressing Bcl2 promoter activation (Additional file 1: Figure S3), and Xbp1 protein (Fig. 3b, Additional file 1: Figure S2) in SP 2/0 cells. This suggests that Gm40600 suppresses Bcl2 transcription by reducing Xbp1 protein levels. Previous studies have shown that Blimp-1 induces expression of Xbp1 [41–43], which promotes antibody production [44, 45]. Our data showed that both Blimp1 and Xbp1 protein levels were reduced in SP 2/0 cells overexpressing Gm40600 (Fig. 3b, Additional file 1: Figure S2). Collectively, these results suggest that Gm40600 induced apoptosis by reducing Blimp1 and Xbp1-mediated Bcl2 transcription.

Blimp1 promotes the survival of PC in healthy donors and MM patients, whereas its deficiency causes PC apoptosis [31, 46]. In accordance with these studies, our data demonstrated that overexpression of Gm40600 reduced both Blimp1 expression (Fig. 3b) and promoted apoptosis (Fig. 1b-d). Thus, Gm40600 overexpression promoted SP 2/0 cell apoptosis by reducing Blimp1 expression.

Finally, we found that Gm40600 expression is positively related to Prdm1 and Xbp1 expression involved in nonmalignant PC generation/maintenance (Table 3). However, Gm40600 overexpression suppressed antibody production in LPS-stimulated primary B cells (Fig. 4). Collectively, these data suggest that Gm40600 is a negative regulator of antibody production.

There are still some unsolved questions: 1, the mechanisms by which Gm40600 reduces Blimp1; 2, the physiological function of Gm40600 in Prdm1 and Xbp1 expression, and nonmalignant PC generation/maintenance; 3, whether there are human orthologs of the Gm40600 gene; 4, the pathogenic role of the human ortholog of Gm40600 gene in MM; 5, whether regulation of the Gm40600 human ortholog can be used to treat MM. Researching these questions will be important for understanding the role of Gm40600 and its human orthologs in MM.

## Conclusions

Compared to LPS-induced PC, cancerous SP 2/0 cells expressed a low level of Gm40600 mRNA. Overexpression of Gm40600 suppressed SP 2/0 cell proliferation and isograft tumor growth by reducing Blimp1 and Xbp1-mediated transcription of the anti-apoptosis molecule Bcl2. Thus, it is necessary to explore the role of a human homolog of Gm40600 in MM. This will guarantee that regulation of a human homolog of Gm40600, or associated factors, may represent a novel therapeutic strategy for treating MM.

## Additional file

Additional file 1 : **Figure S1.** Gm40600 mRNA expression was significantly lower in stable Gm40600-expressing SP 2/0 cells than that in LPS-induced plasmablasts/plasma cells (PB/PC). B cells from the splenocytes of three 8–9-week-old Balb/c mice per group were sorted by B220 microbeads, and stimulated for 3 days in vitro by 10 μg/ml LPS. The stable Vector- or Gm40600-expressing SP 2/0 cells were thawed, passaged for 3 times and then cultured for 3 days in fresh medium. Gm40600 mRNA expression was determined by qPCR in Vector-expressing SP 2/0 cells (Vector-SP2/0), Gm40600-expressing SP 2/0 cells (Gm40600-SP2/0) and LPS-stimulated PB/PC (LPS-PB/PC) were determined. Relative mRNA levels are normalized to GAPDH mRNA expression and calculated relative to the mRNA expression in SP 2/0 cells, set to 1. **Figure S2.** Gm40600 overexpression reduced Blimp1 and Xbp-1 protein in SP 2/0 cells. Vector- or Gm40600-expressing SP 2/0 cells were thawed, passaged for 3 times and then cultured for 3 days in fresh medium. The cells were then collected and subjected to immunoblot analysis with monoclonal anti-mouse antibodies for Xbp1, Blimp1, and β-tubulin. Results represent three independent experiments. **Figure S3.** Gm40600 overexpression suppressed the Bcl2 promoter activation in SP 2/0 cells. The luciferase reporter vector pEZX-PG04.1/Bcl2 promoter (− 1323 ~ + 160 bp) and renilla luciferase reporter vector pRLSV-40 vector were co-transduced into stable Gm40600- or vector-expressing SP 2/0 cells. The cells were cultured for 3 days. Dual luciferase reporter gene expression was analyzed, and the results are shown as the ratio of firefly to renilla luciferase activity. The data represent three independent experiments. Error bars, SEM. Two tailed Student’s t-test, **P < 0.01. (PDF 203 kb)

## Data Availability

The generated and/or analyzed datasets of the current study are available in the ArrayExpress repository, http://www.ebi.ac.uk/arrayexpress/experiments/E-MTAB-7136.

## Abbreviations

CCK8: Cell counting kit-8
CSR: Class switch recombination
FACS: Fluorescence-activated cell sorting
Ig: Immunoglobulin
LPS: Lipopolysaccharide
MM: Multiple myeloma
PBs: Plasmablasts
PCs: Plasma cells
SHM: Somatic hypermutation
