# Supplementary material for: Gm40600 suppressed SP 2/0 isograft tumor by reducing Blimp1 and Xbp1 proteins
Source: BMC Cancer. 2019 Jul 16;19:700. doi: 10.1186/s12885-019-5848-1 (PMC6636126; doi:10.1186/s12885-019-5848-1)
Supplement: Supplementary file 1 — : Figure S1. Gm40600 mRNA expression was significantly lower in stable Gm40600-expressing SP 2/0 cells than that in LPS-induced plasmablasts/plasma cells (PB/PC). B cells from the splenocytes of three 8–9-week-old Balb/c mice per group were sorted by B220 microbeads, and stimulated for 3 days in vitro by 10 μg/ml LPS. The stable Vector- or Gm40600-expressing SP 2/0 cells were thawed, passaged for 3 times and then cultured for 3 days in fresh medium. Gm40600 mRNA expression was determined by qPCR in Vector-expressing SP 2/0 cells (Vector-SP2/0), Gm40600-expressing SP 2/0 cells (Gm40600-SP2/0) and LPS-stimulated PB/PC (LPS-PB/PC) were determined. Relative mRNA levels are normalized to GAPDH mRNA expression and calculated relative to the mRNA expression in SP 2/0 cells, set to 1. Figure S2. Gm40600 overexpression reduced Blimp1 and Xbp-1 protein in SP 2/0 cells. Vector- or Gm40600-expressing SP 2/0 cells were thawed, passaged for 3 times and then cultured for 3 days in fresh medium. The cells were then collected and subjected to immunoblot analysis with monoclonal anti-mouse antibodies for Xbp1, Blimp1, and β-tubulin. Results represent three independent experiments. Figure S3. Gm40600 overexpression suppressed the Bcl2 promoter activation in SP 2/0 cells. The luciferase reporter vector pEZX-PG04.1/Bcl2 promoter (− 1323 ~ + 160 bp) and renilla luciferase reporter vector pRLSV-40 vector were co-transduced into stable Gm40600- or vector-expressing SP 2/0 cells. The cells were cultured for 3 days. Dual luciferase reporter gene expression was analyzed, and the results are shown as the ratio of firefly to renilla luciferase activity. The data represent three independent experiments. Error bars, SEM. Two tailed Student’s t-test, **P < 0.01. (PDF 203 kb) [file 12885_2019_5848_MOESM1_ESM.pdf]

Figure S1.

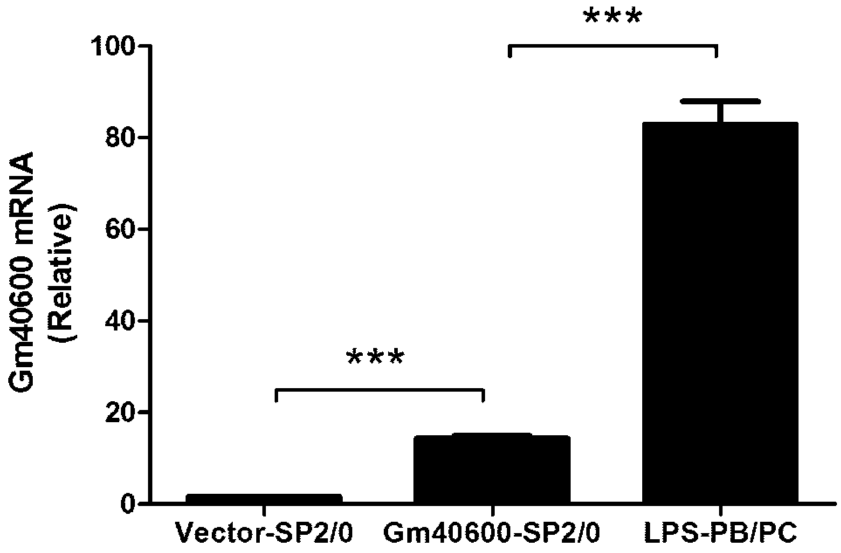

Figure S1. Gm40600 mRNA expression was significantly lower in stable Gm40600-expressing SP 2/0 cells than that in LPS-induced plasmablasts/plasma cells (PB/PC). B cells from the splenocytes of three 8-9-week-old Balb/c mice per group were sorted by B220 microbeads, and stimulated for 3 days *in vitro* by 10 μg/ml LPS. The stable Vector or Gm40600-expressing SP 2/0 cells were thawed, passaged for 3 times and then cultured for 3 days in fresh medium. Gm40600 mRNA expression was determined by qPCR in Vector-expressing SP 2/0 cells (Vector-SP2/0), Gm40600-expressing SP 2/0 cells (Gm40600-SP2/0) and LPS-stimulated PB/PC (LPS-PB/PC) were determined. Relative mRNA levels are normalized to GAPDH mRNA expression and calculated relative to the mRNA expression in SP 2/0 cells, set to 1.

Figure S2.

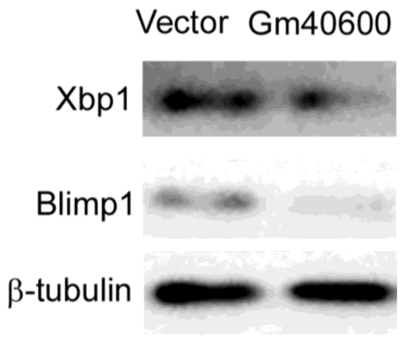

Figure S2. Gm40600 overexpression reduced Blimp1 and Xbp-1 protein in SP 2/0 cells. Vector- or Gm40600-expressing SP 2/0 cells were thawed, passaged for 3 times and then cultured for 3 days in fresh medium. The cells were then collected and subjected to immunoblot analysis with monoclonal anti-mouse antibodies for Xbp1, Blimp1, and β-tubulin. Results represent three independent experiments.

Figure S3.

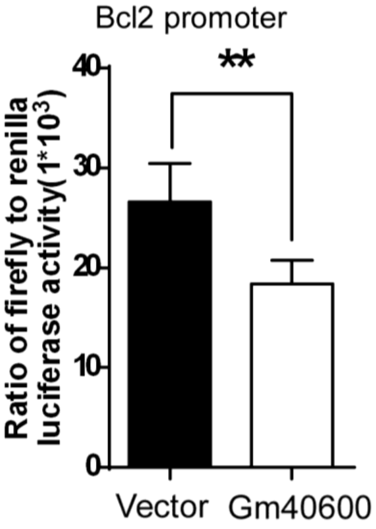

Figure S3. Gm40600 overexpression suppressed the Bcl2 promoter activation in SP 2/0 cells. The luciferase reporter vector pEZX-PG04.1/Bcl2 promoter (− 1323 ~ + 160 bp) and renilla luciferase reporter vector pRLSV-40 vector were co-transduced into stable Gm40600- or vector-expressing SP 2/0 cells. The cells were cultured for 3 days. Dual luciferase reporter gene expression was analyzed, and the results are shown as the ratio of firefly to renilla luciferase activity. The data represent three independent experiments. Error bars, SEM. Two tailed Student's t-test, **P < 0.01.
